# Oligomeric states in sodium ion-dependent regulation of cyanobacterial histidine kinase-2

**DOI:** 10.1007/s00709-017-1196-7

**Published:** 2017-12-30

**Authors:** Iskander M. Ibrahim, Liang Wang, Sujith Puthiyaveetil, Norbert Krauß, Jon Nield, John F. Allen

**Affiliations:** 1 Department of Biochemistry and Purdue Center for Plant Biology, Purdue University, West Lafayette, IN USA; 2 School of Biological and Chemical Sciences, Queen Mary University of London, London, UK; 3 Botanisches Institut, Karlsruher Institut für Technologie, Karlsruhe, Germany; 4 Research Department of Genetics, Evolution and Environment, University College London, London, UK

**Keywords:** Two-component regulatory system, Sensor histidine kinase, Oligomerisation, Size-exclusion chromatography, Single-particle electron microscopy, Transcriptional regulation, Protein phosphorylation

## Abstract

Two-component signal transduction systems (TCSs) consist of sensor histidine kinases and response regulators. TCSs mediate adaptation to environmental changes in bacteria, plants, fungi and protists. Histidine kinase 2 (Hik2) is a sensor histidine kinase found in all known cyanobacteria and as chloroplast sensor kinase in eukaryotic algae and plants. Sodium ions have been shown to inhibit the autophosphorylation activity of Hik2 that precedes phosphoryl transfer to response regulators, but the mechanism of inhibition has not been determined. We report on the mechanism of Hik2 activation and inactivation probed by chemical cross-linking and size exclusion chromatography together with direct visualisation of the kinase using negative-stain transmission electron microscopy of single particles. We show that the functional form of Hik2 is a higher-order oligomer such as a hexamer or octamer. Increased NaCl concentration converts the active hexamer into an inactive tetramer. The action of NaCl appears to be confined to the Hik2 kinase domain.

## Introduction

**Fig. 1:**
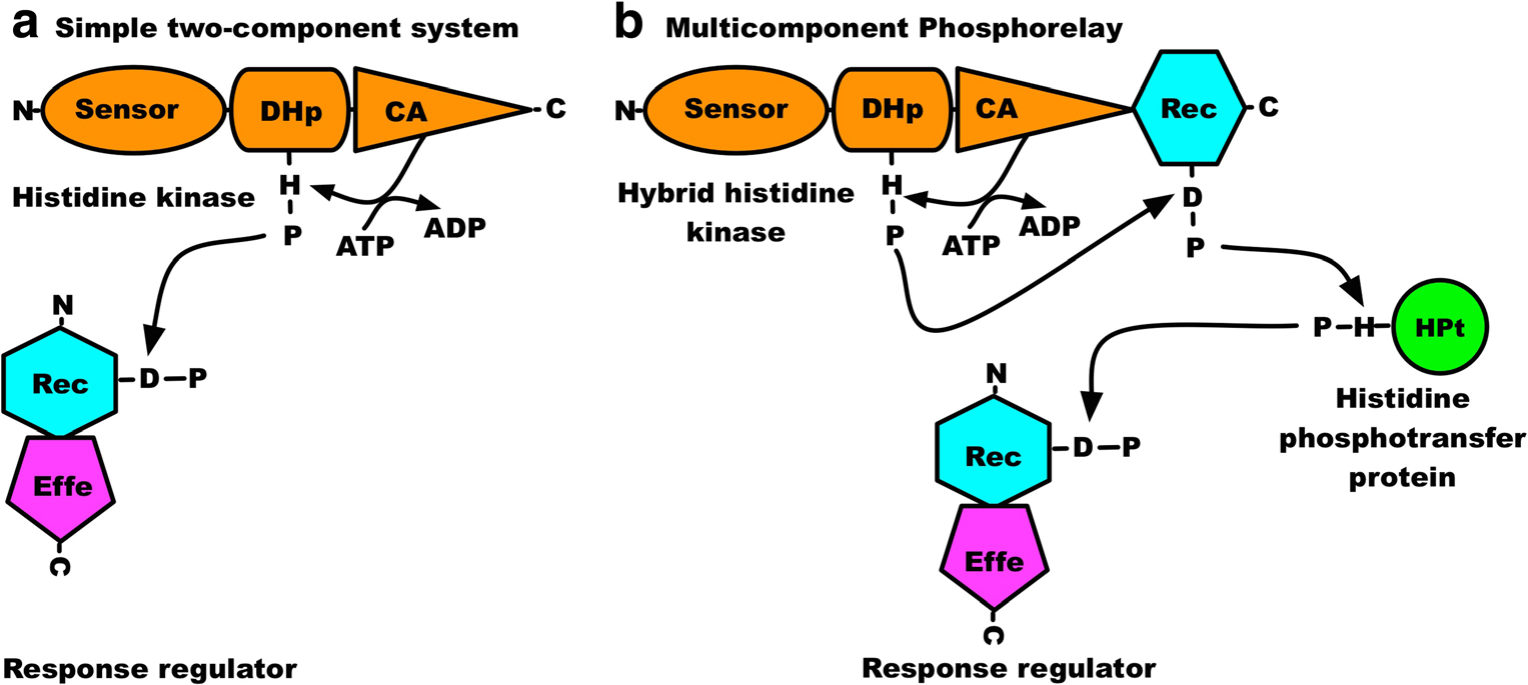
Domain architecture of two-component systems. **a** A simple two-component system. **b** A multicomponent phosphorelay. The sensor domain is indicated by an oval, the dimerisation and phosphoaccepting (DHp) domain by a cylinder, the catalytic and ATP-binding (CA) domain by a triangle, the receiver (Rec) domain by a hexagon, the effector (Effe) domain by a pentagon, and the histidine phosphotransfer protein (HPt) by a circle. The amino and the carboxy termini are shown as N and C, respectively (Adapted from Ibrahim 2013)

Bacteria, algae, plants and fungi adapt to changes in their environments and often utilise a sensor-response circuit known as a two-component signal transduction system (TCS) to elicit physiological responses. TCSs are particularly diverse and widely distributed in bacteria (Skerker et al. 2008; Stock et al. 2000). The simplest form of a TCS consists of just two proteins: a conserved sensor histidine kinase (component 1) and a response regulator (component 2) (Fig. 1a).

A signal transduction cascade in bacteria usually begins at the cell membrane from where the signal propagates to the cytoplasm through the transmembrane domain of a sensor histidine kinase. The environmental cue is detected by the sensor domain located near the N terminus of the histidine kinase polypeptide. In contrast, some sensor histidine kinases exist as soluble cytoplasmic or cytosolic proteins that perceive changes within the cell without direct membrane attachment. Functional forms of both membrane-anchored and soluble histidine kinases occur predominantly as homodimers that contain conserved dimerisation and phosphotransfer (DHp) and catalytic and ATP-binding (CA) domains (Fig. 1). A higher-order oligomeric state, the tetramer, is promoted by an intermolecular disulphide bond in the histidine kinases DcuS (dicarboxylate uptake sensor and regulator) (Scheu et al. 2010), RegB (regulator B) (Swem et al. 2003), AtoS (sensor kinase-controlling ornithine decarboxylase antizyme) (Filippou et al. 2008) and KdpD (osmosensitive potassium channel sensor histidine kinase) (Heermann et al. 1998). Formation of the higher-order oligomer appears to silence their autophosphorylation. In contrast, the well-characterised membrane-extrinsic, soluble histidine kinases EnvZc (core kinase domain of EnvZ) (Cai et al. 2003), VirAc (virulence A) (Pan et al. 1993), and CheA (chemotaxis histidine kinase A) (Surette et al. 1996) are reported to exist solely as inactive monomers or as active dimers. Proteins of the phytochrome superfamily are photoreceptors widely distributed in both prokaryotes (Kacprzak et al. 2017; Nagano et al. 2016; Scheerer et al. 2010) and eukaryotes (Duanmu et al. 2014; Hirose et al. 2013; Rockwell and Lagarias 2017). Phytochromes are members of the class of soluble histidine kinases that form stable homodimers (Noack et al. 2007).

An in vitro study with the CheA sensor kinase indicates that its oligomeric state is concentration-dependent. CheA exists at low protein concentrations predominantly as an inactive monomer while the extent of its dimerisation increases with increasing protein concentration (Surette et al. 1996). Thus, CheA is likely to exist in vivo at equilibrium between its inactive monomeric and active dimeric forms, with its interaction with a ligand acting as a signal that shifts this equilibrium towards the active dimer. In contrast, the membrane-anchored sensor kinase DcuS from *Escherichia coli* exists as monomer, dimer and tetramer both in vitro and in vivo (Scheu et al. 2010). The ArcB sensor kinase of *E. coli* contains two conserved redox-active cysteines that are regulated by the redox state of ubiquinone. Oxidation of these cysteines leads to intermolecular disulphide bond formation between two monomers of ArcB, locking it into an inactive tetrameric state as a protein kinase (Georgellis et al. 2001; Malpica et al. 2004). The RegB histidine kinase of purple photosynthetic bacteria is also converted from an active dimer to an inactive tetramer by oxidation of its conserved cysteine (Swem et al. 2003).

Histidine kinase 2 (Hik2) is one of the three conserved and apparently complete histidine kinases found in cyanobacteria (Ashby and Houmard 2006). The closest Hik2 homologue in algae and higher plants is CSK (chloroplast sensor kinase) (Puthiyaveetil et al. 2008). Chloroplast two-component systems linking photosynthesis with gene transcription are derived from those of cyanobacteria (Puthiyaveetil and Allen 2009), and their function in redox regulation of target genes may account for the persistence, in evolution, of chloroplast DNA (Allen 1993a; Allen 2015; Allen 2017). A recombinant cyanobacterial Hik2 protein undergoes autophosphorylation on its conserved histidine residue and transfers the phosphoryl group to response regulators Rre1 and RppA (Ibrahim et al. 2016a). Rre1 is also modulated by Hik34 in response to increased temperature (Kobayashi et al. 2017).

In its unmodified state, Hik2 appears to be autokinase-active, and treatment with Na^+^ ions abolishes its autophosphorylation (Ibrahim et al. 2016a). The exact mechanism by which the activity of Hik2 is switched off by Na^+^ ions is not yet clear. Here, we determine the mechanism of Hik2 activation and inactivation using chemical cross-linking and size-exclusion chromatography, together with direct visualisation of the kinase using negative-stain transmission electron microscopy of single particles. We show that Hik2 is present in multiple oligomeric states in vitro and that a signal such as Na^+^ converts higher oligomers into a tetramer, thus inactivating it as the protein kinase that otherwise donates the phosphoryl group to its response regulators.

## Materials and methods

### Construction of recombinant plasmids

Coding sequences were cloned using the primer pairs listed in Table 1. These correspond to the following: the full-length *Synechocystis* sp. PCC6803 Hik2 (slr1147) and the *Thermosynechococcus elongatus* BP-1 (tlr0195) full-length kinase domain corresponding to amino acids 142–386 and the DHp domain corresponding to amino acids 142–270. PCR products were digested with *NdeI* and *SalI* endonucleases (New England BioLabs) and cloned into pET-21b (Novagen) expression vector digested with *NdeI* and *XhoI*. The identities of the recombinant clones were confirmed by sequencing (results not shown).

**Table 1:** Primers used for Hik2 cloning

– Synecho_Hik2 (cloned in pET-21b) forward: GCGCGCcatatgGCCGGTTCCATCTCA reverse: GCGCGCctcgagCACTTGTTCTCCAGAGCG
– Thermo_Hik2F (cloned in pET-21b) forward: GCGCcatatgATGCTCTGGCCAGCCAGT reverse: GCGCGCgtcgacTGGTTCCACCTTCATTTG
– Thermo_Hik2T (cloned in pET-21b) forward: GCGCcatatgATGCACTCCCCTGCCCAGCCA reverse: GCGCGCgtcgacTGGTTCCACCTTCATTTG
– Thermo_DHp (cloned in pET-21b) forward: GCGCcatatgATGCACTCCCCTGCCCAGCCA reverse: GCGCgtcgacTTCCTCGAGCCAGATCGG

Sequences in lowercase are restriction-site overhangs.

### Expression and purification of recombinant Hik2

Recombinant plasmids were transformed into *E. coli* BL21(DE3) chemically competent cells (Stratagene). Transformed bacterial colonies, grown on agar plates, were used to inoculate starter cultures (10 mL each) in Luria broth (LB) growth media (Sambrook et al. 1989) with 100 μg mL^−1^ ampicillin as the selectable marker. Each culture was grown overnight, diluted 1:100 in 1 L of LB media, and then grown at 37 °C to an optical density at 600 nm of ~ 0.55 before inducing protein expression with 0.5 mM isopropyl β-d-1-thiogalactopyranoside (IPTG) (Melford). Bacterial cultures were then grown for a further 16 h at 16 °C. Cells were harvested by centrifugation at 9000×*g* for 10 min at 4 °C. The pellet was suspended in a buffer containing 300 mM NaCl, 20 mM Tris-HCl adjusted to pH 7.4, 25 mM imidazole and 1 mM PMSF, and the cells were then lysed with an EmulsiFlex-C3 homogeniser (Avestin). The lysate was separated by centrifugation at 39000×*g* for 20 min at 4 °C. The supernatant was applied to a Ni^2+^ affinity chromatography column (GE Healthcare), and the proteins were purified according to the column manufacturer’s instructions.

### Size-exclusion chromatography

The oligomeric states of Hik2 were determined by subjecting the purified proteins to Superdex 200 10/300GL chromatography (GE Healthcare), equilibrated with 20 mM Tris-HCl (pH 7.6) and 10 mM NaCl (low salt) or with 20 mM Tris-HCl (pH 7.6) and 500 mM NaCl (high salt). Calibration curves were obtained as above at low or high salt concentrations using standard proteins of known molecular mass: apoferritin (443 kDa), alcohol dehydrogenase (150 kDa) and carbonic anhydrase (29 kDa). Blue dextran (2000 kDa) was used to determine the void volume (Vo). The molecular masses of Hik2 were determined by using the calibration curve at 10 mM NaCl for the low salt condition and the calibration curve at 500 mM NaCl for high salt.

### Chemical cross-linking

The full-length Hik2 protein was desalted into cross-linking reaction buffer (25 mM HEPES-NaOH at pH 7.5, 5 mM KCl and 5 mM MgCl_2_) using a PD-10 desalting column (Amersham Biosciences). Chemical cross-linking was carried out in a total reaction volume of 20 μL in cross-linking reaction buffer containing desalted Hik2 protein at concentrations of 2, 3, 4, 5, 10, 15, 20, 25, 30, 35, 40, 45 or 50 μM. The cross-linking agent dithiobis(succinimidylpropionate) (DSP) (Lomant and Fairbanks 1976) was added from 24.73 mM stock solution in dimethyl sulphoxide to give a final DSP concentration of 2 mM. Reactions were incubated at 23 °C for 10 min. Reactions were stopped by the addition of a solution containing 50 mM Tris-HCl and 10 mM glycine giving pH 7.5. The above reaction was repeated with 2 μM Hik2 and varying concentrations of DSP. Two micrograms of cross-linked proteins was resolved upon 10% SDS-PAGE (sodium dodecyl sulphate polyacrylamide gel electrophoresis), and the gel was stained with Coomassie brilliant blue.

### In vitro autophosphorylation

Autophosphorylation was performed with 2 μM of purified recombinant Hik2 protein in a kinase reaction buffer (50 mM Tris-HCl at pH 7.5, 50 mM KCl, 10% glycerol and 10 mM MgCl_2_) in a final reaction volume of 25 μL. The autophosphorylation reaction was initiated by the addition of 5 μL of a solution containing 2.5 mM disodium ATP (Sigma) with 2.5 μCi [γ-^32^P]-ATP (6000 Ci mmol^−1^) (PerkinElmer). Reactions were incubated for 15 s at 22 °C. Cross-linking was performed as above except that the autophosphorylation reaction was terminated, in this case, by the addition of 6 μL of fivefold-concentrated non-reducing Laemmli sample buffer (Laemmli 1970). Reaction products were resolved using a 12% non-reducing SDS-PAGE gel. The gel was rinsed with SDS running buffer and transferred into a polyethylene bag. The sealed bag was exposed to a phosphor plate overnight. The incorporated γ-^32^P was visualised using autoradiography.

### Sequence analysis

Sequence similarity search was carried out with blastP and blastn (Altschul et al. 1990) using the public databases Cyanobase (http://genome.kazusa.or.jp/cyanobase) and Joint Genome Institute (JGI) (http://www.jgi.doe.gov/). Domain prediction was carried out using the SMART database (http://smart.embl-heidelberg.de/smart/set_mode.cgi?NORMAL=1) (Schultz et al. 1998).

### Transmission electron microscopy and single-particle analyses

Five independent Hik2 samples were subjected in turn to a dilution series, applied to carbon-coated (thin-layer) copper 300-mesh EM grids (Agar, Ltd.) and negatively stained using freshly prepared 2% uranyl acetate. A protein concentration that ensured an even spread of single particles over the carbon film surface was found for each sample and dilution. Two hundred one micrographs (each being 2672 × 2672 pixels) were recorded using an Olympus Morada CCD camera system attached to a JEOL model 1230 TEM equipped with a tungsten filament and operating at × 80,000 magnification and 80 keV. This gave a sampling frequency of 5.962 Å per pixel at the specimen scale; however, a limitation of ~ 15-Å resolution is expected to result from the presence of the negative stain. The Fourier space power spectrum was calculated for each micrograph, and 41 micrographs were chosen for further single-particle analysis on the basis that they displayed minimal drift and astigmatism and had a first minimum at better than ~ 17-Å resolution. Single-particle complexes were floated out into boxes of 64 × 64 pixels in size. Given that no correction was applied for the contrast transfer function (CTF), the final class averages were low band-pass filtered to ~ 20-Å resolution. Initial single particle images were selected using the ‘boxer’ module of EMAN2 (Tang et al. 2007), with the boxing algorithm directed to pick automatically all possible single particles present, but not to band-pass or normalise. The Imagic-5 software environment (van Heel et al. 1996) was then used for image normalisation, band-pass filtering, reference-free alignment and multivariate statistical classification of the single-particle image data set.

## Results

### Distribution of Hik2 and domain architecture

**Fig. 2:**
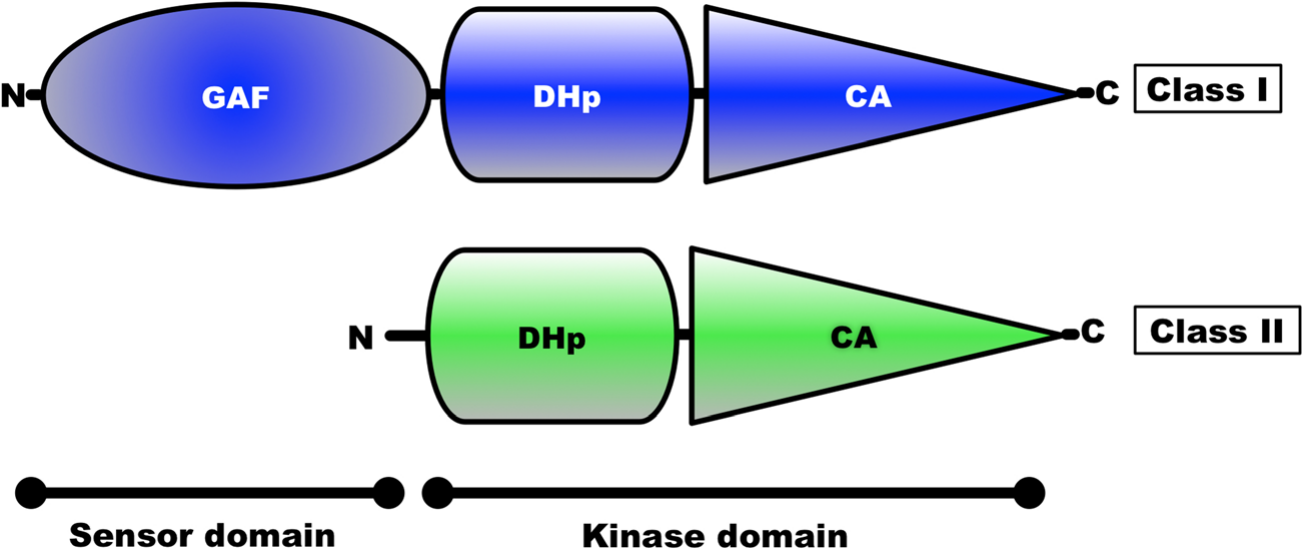
Domain architecture of Hik2 proteins. The amino and the carboxy termini are shown as N and C, respectively. The domain architecture of Hik2 was predicted using the SMART database (Schultz et al. 1998). The predicted sensor domain is shown as GAF. The kinase core contains the DHp and CA domains. The colour corresponds to different forms of Hik2 proteins, i.e. blue representing the full-length, class I Hik2 protein and green representing class II Hik2 protein

Histidine kinases contain a conserved kinase core domain and a variable sensor domain. The kinase core domain is essential for autokinase and phosphotransfer activities. The Hik2 homologue that is present in almost all cyanobacteria and plants contains a conserved kinase core domain consisting of DHp and CA together with a GAF sensor domain (Ibrahim et al. 2016a; Puthiyaveetil et al. 2008). In three cyanobacterial species Hik2 is present as a truncated form without a GAF sensor domain (Ashby and Houmard 2006). We have examined the domain architecture and distribution of Hik2 homologues in cyanobacteria and chloroplasts. Hik2 domain architecture revealed that there are two forms of the Hik2 proteins in cyanobacteria and chloroplasts. Here, these forms are designated class I and class II (Table 2 and Fig. 2). Class I Hik2 proteins contain the full-length GAF domain as predicted with SMART database. Class II Hik2 proteins are predicted to have no GAF domain. As shown in Table 2, most cyanobacteria and chloroplasts have a class I Hik2. The least widely distributed Hik2 is the class II protein that is found only in three cyanobacteria (Ashby and Houmard 2006).

**Table 2:**
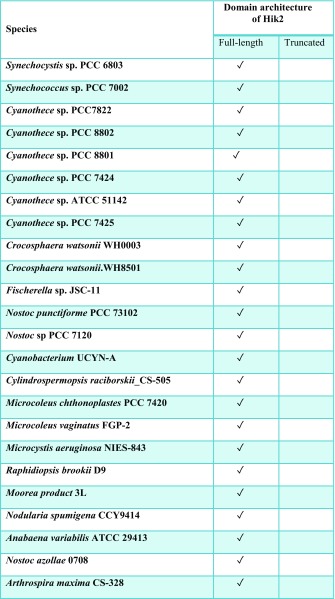

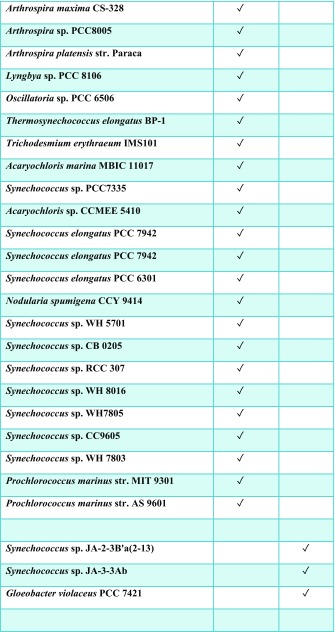

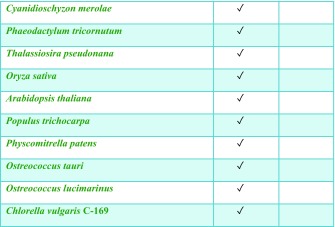
Distribution of the full-length and truncated forms of Hik2. Tick mark indicates the presence of Hik2

### Recombinant protein production

**Fig. 3:**
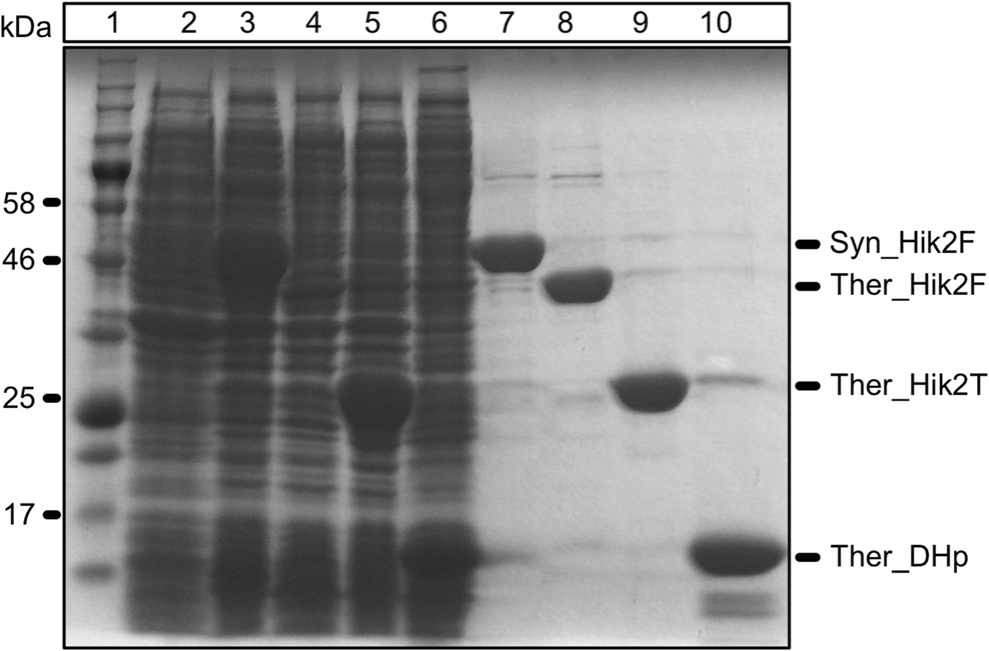
Protein overexpression and purification. The full-length *Synechocystis* sp. PCC 6803 and *Thermosynechococcus elongatus* BP-1 Hik2 and the truncated form of *Thermosynechococcus elongatus* BP-1 were overexpressed and purified as described in the ‘Materials and methods’ section. The following samples were loaded on a 10% SDS-PAGE. Lane 1 is the protein molecular mass marker; lane 2 is the total cell fraction before IPTG induction; lanes 3–6 are the total cell fractions after IPTG induction: lane 3, full-length *Synechocystis* Hik2 (Syn_Hik2F); lane 4, *Thermosynechococcus elongatus* BP-1 full-length Hik2 (Ther_Hik2F); lane 5, *Thermosynechococcus* truncated form (Ther_Hik2T); lane 6, *Thermosynechococcus* DHp domain (Ther_DHp); lanes 7–10 correspond to the elution fractions from the Ni^2+^ affinity chromatography column: lane 7, Syn_Hik2F; lane 8, Ther_Hik2F; lane 9, Ther_Hik2T; lane 10, Ther_DHp. The positions of the overexpressed proteins are indicated on the right, and the molecular masses of selected marker proteins are indicated on the left

The following proteins were cloned, overexpressed and purified from *E. coli*: full-length *Synechocystis* sp. PCC 6803 (Syn_Hik2F), full-length *Thermosynechococcus elongatus* BP-1 Hik2 (Ther_Hik2F), the truncated core kinase domain of *T. elongatus* BP-1 protein containing only the DHp and CA subdomains (Ther_Hik2T), and the truncated DHp domain of *T. elongatus* BP-1 (Ther_DHp). Figure 3 lane 7 shows the purified Syn_Hik2F; lane 8, Ther_Hik2F; lane 9, Ther_Hik2T; and lane 10, Ther_DHp. The calculated molecular masses are as follows: Syn_Hik2F, 48.5 kDa; Ther_Hik2F, 44.2 kDa; Ther_Hik2T, 26.2 kDa; and Ther_DHp, 15.1 kDa. The apparent molecular masses on the SDS-PAGE are as follows: Syn_Hik2F, 50 kDa; Ther_Hik2F, 45 kDa; Ther_Hik2T, 29 kDa; and Ther_DHp, 16 kDa.

### Hik2 exists in higher-order oligomeric states

An earlier study has shown that sodium ions inhibit the autophosphorylation activity of full-length Hik2 (Ibrahim et al. 2016a). In order to elucidate its salt-sensing mechanism, we investigated the oligomeric state of Hik2 in a buffer supplemented with or lacking NaCl. The oligomeric state of Hik2 was explored by size-exclusion chromatography and chemical cross-linking with DSP. These two techniques were chosen because size-exclusion chromatography is the most suited for stable protein-protein interactions, while DSP provides a more direct method of studying transient semi-stable protein-protein interactions. DSP is a symmetric molecule with two reactive groups connected by a spacer arm that is 12 Å in length. Thus, DSP can form amide bonds with amino groups of two polypeptides that are in close proximity, for example, between two monomers in a dimer or between two dimers in a tetramer. DSP links polypeptides that interact under physiological conditions and therefore has advantages for studying oligomeric states of semi-stable protein-protein interactions.

**Fig. 4:**
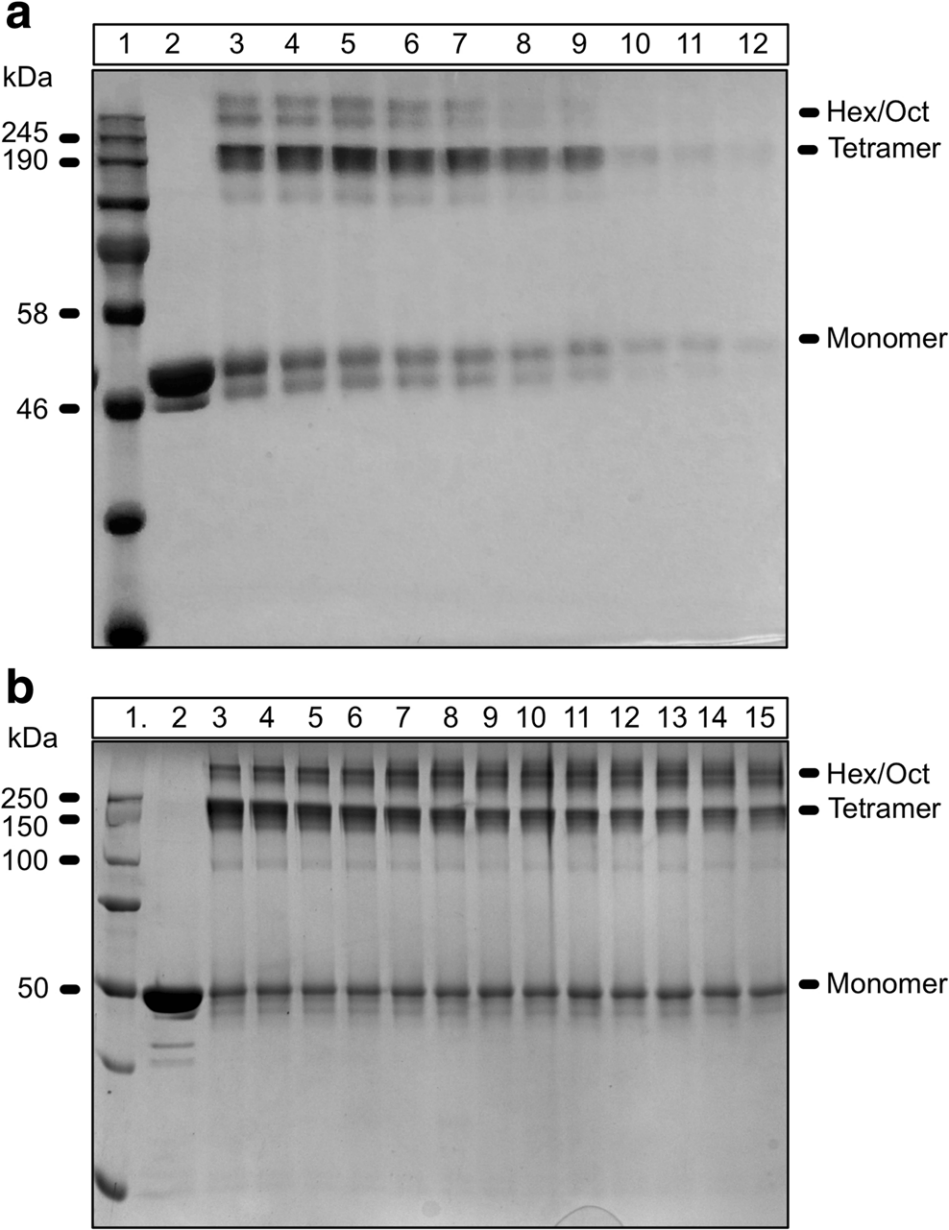
Effects of chemical cross-linking and protein concentration on the oligomeric state of *Synechocystis* sp. PCC 6803 Hik2. **a** Chemical cross-linking. Lane 1 shows the protein molecular mass markers; lane 2, the untreated Hik2 protein (control); lane 3, the Hik2 treated with 1 mM DSP; lane 4, the Hik2 treated with 2 mM DSP; lane 5, the Hik2 treated with 3 mM DSP; lane 6, the Hik2 treated with 4 mM DSP; lane 7, the Hik2 treated with 5 mM DSP; lane 8, the Hik2 treated with 6 mM DSP; lane 9, the Hik2 treated with 7 mM DSP; lane 10, the Hik2 treated with 9 mM DSP; lane 11, the Hik2 treated with 10 mM DSP; and lane 12, the Hik2 treated with 11 mM DSP. Samples were subjected to non-reducing 10% SDS-PAGE. The molecular masses are shown on the left in kilodalton. The oligomeric states of Hik2 are indicated on the right. **b** Protein concentration. Lane 1 shows the protein molecular mass markers. Lane 2, 2 μM untreated Hik2 protein. Proteins in the following lanes were cross-linked at the following protein concentrations: lane 3, 2 μM; lane 4, 3 μM; lane 5, 4 μM; lane 6, 5 μM; lane 7, 10 μM; lane 8, 15 μM; lane 9, 20 μM; lane 10, 25 μM; lane 11, 30 μM; lane 12, 35 μM; lane 13, 40 μM; lane 14, 45 μM; and lane 15, 50 μM. Molecular masses are shown on the left-hand side in kilodalton. Different oligomeric states are labelled on the right-hand side of the gel

In order to determine whether the oligomeric state of Hik2 is dependent on DSP concentration, Hik2 proteins were incubated with different concentrations of the cross-linker DSP for 10 min at 23 °C. Cross-linked products were then resolved on a non-reducing SDS-PAGE. Figure 4a lane 2 shows that the untreated Hik2 protein migrated on a non-reducing SDS-PAGE with an apparent molecular mass of 50 kDa, corresponding to its monomeric form. Figure 4a lanes 3–10 indicate that chemical cross-linking produced four distinct protein bands at apparent molecular masses corresponding approximately to multiples of 50 kDa. The first band at 50 kDa can be assigned to the monomer, a second band just above 190 kDa to a tetramer, and two further bands above 250 kDa to higher oligomers, possibly a hexameric and an octameric form. Although increasing the concentration of DSP from 1 to 3 mM had no effect on the oligomeric state of Hik2 (Fig. 4a, lanes 3–5), increasing the concentration above 3 mM resulted in a decrease in both monomeric and higher-order oligomers (Fig. 4a, lanes 6–12) and therefore, only 2 mM of DSP was used in subsequent experiments.

It has been shown that dimerisation of CheA is concentration-dependent (Surette et al. 1996). We, therefore, examined whether the oligomeric state of Hik2 depends on its concentration. Cross-linking was performed with differing concentrations of Hik2 proteins, ranging from 2 to 50 μM, while the concentration of DSP, incubation times and temperature were kept constant. Equal quantities of cross-linked Hik2 proteins were then analysed with non-reducing SDS-PAGE. No correlation is observed between the apparent quantity of the monomeric state of Hik2 and protein concentration while the quantity of the tetramer appears to decrease and hexamer and octamer increased with increasing protein concentration (Fig. 4b).

### The monomer, tetramer and hexamer forms of Hik2 are autokinase-active, and salt converts the higher-order oligomers into a tetramer

**Fig. 5:**
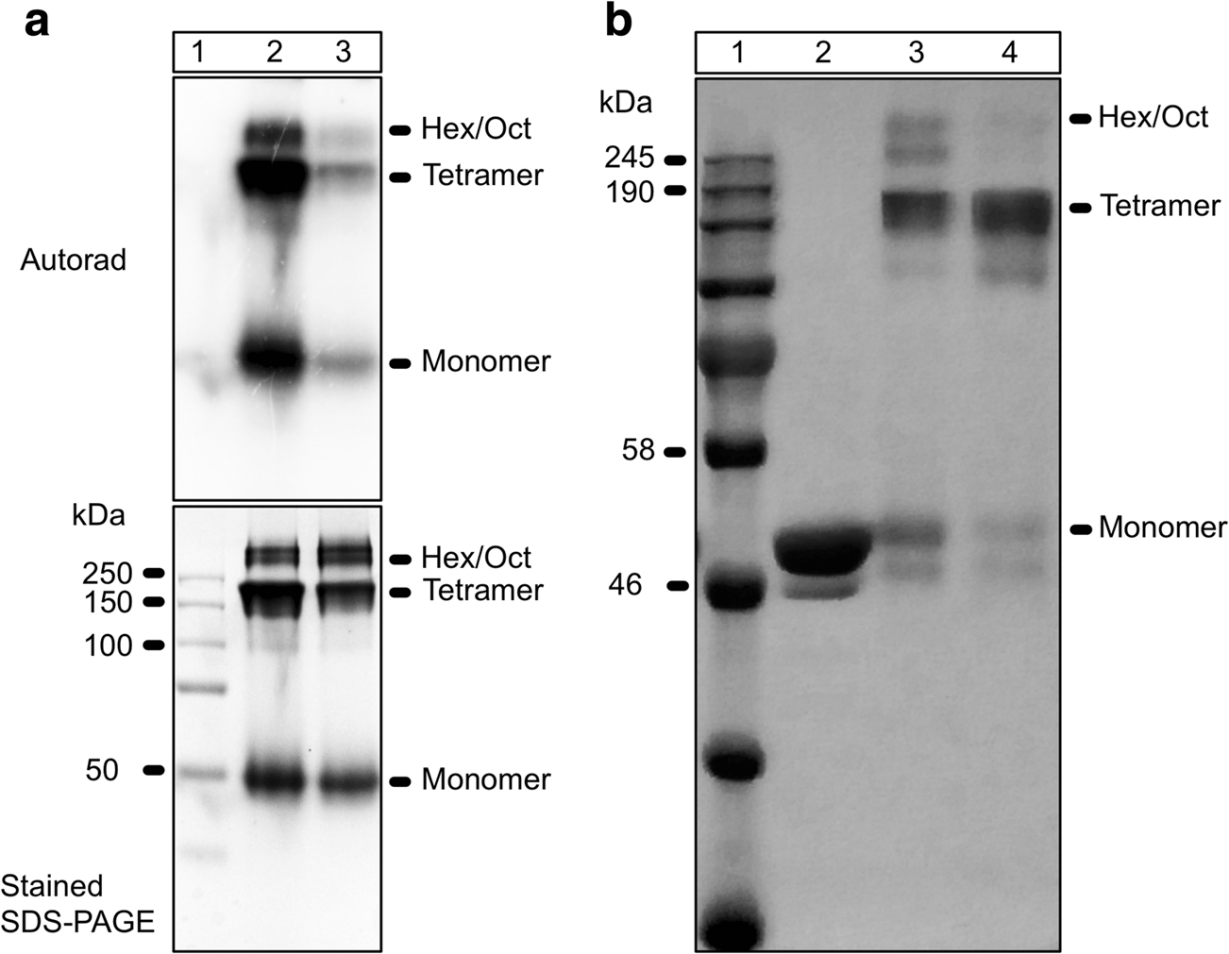
Functional characterisation of oligomeric states of *Synechocystis* sp. PCC 6803 Hik2. **a** Autophosphorylation activity of Hik2. Lane 1 shows the protein molecular mass markers; lane 2, the Hik2 protein that was allowed to autophosphorylate before cross-linking; and lane 3, the Hik2 protein that was first cross-linked, followed by autophosphorylation. **b** Effect of salt on the oligomeric state of Hik2. Lane 1 shows the protein molecular mass markers; lane 2, the untreated Hik2 protein (control); lane 3, salt-untreated cross-linked Hik2; and lane 4, the salt-treated cross-linked Hik2. Different oligomeric states are labelled on the right-hand side of the gel

In order to investigate the functional states of Hik2 oligomers, we carried out autophosphorylation of Hik2 before and after it was cross-linked. Figure 5a, lane 2, shows a Hik2 protein that was allowed to autophosphorylate and then cross-linked with DSP. The result was phosphorylated monomeric, tetrameric and higher-order oligomeric forms. Figure 5a, lane 3, shows that Hik2 protein, which was first cross-linked and then subjected to the autophosphorylation assay, produced monomers, tetramers and higher oligomers relatively inactive in autophosphorylation. Thus, cross-linking may lock the protein into an inactive state. Since the activity of Hik2 is suppressed by salt (Ibrahim et al. 2016a), we then investigated the effect of salt on the oligomeric state of Hik2. Figure 5b, lane 2, shows NaCl-untreated and non-cross-linked Hik2 protein migrating with the apparent molecular mass of a monomer, and in lane 3, NaCl-untreated but cross-linked protein migrating as monomers, tetramers and higher-order oligomers as expected. Lane 4 shows a Hik2 that was treated with NaCl first and then cross-linked. Addition of NaCl appears to have resulted in the conversion of the hexamers and octamers into tetramers. The proportion of the monomeric form is also decreased in Fig. 5b lane 4 in the salt-treated sample.

### NaCl converts the active hexamer form of Hik2 into a tetramer

**Fig. 6:**
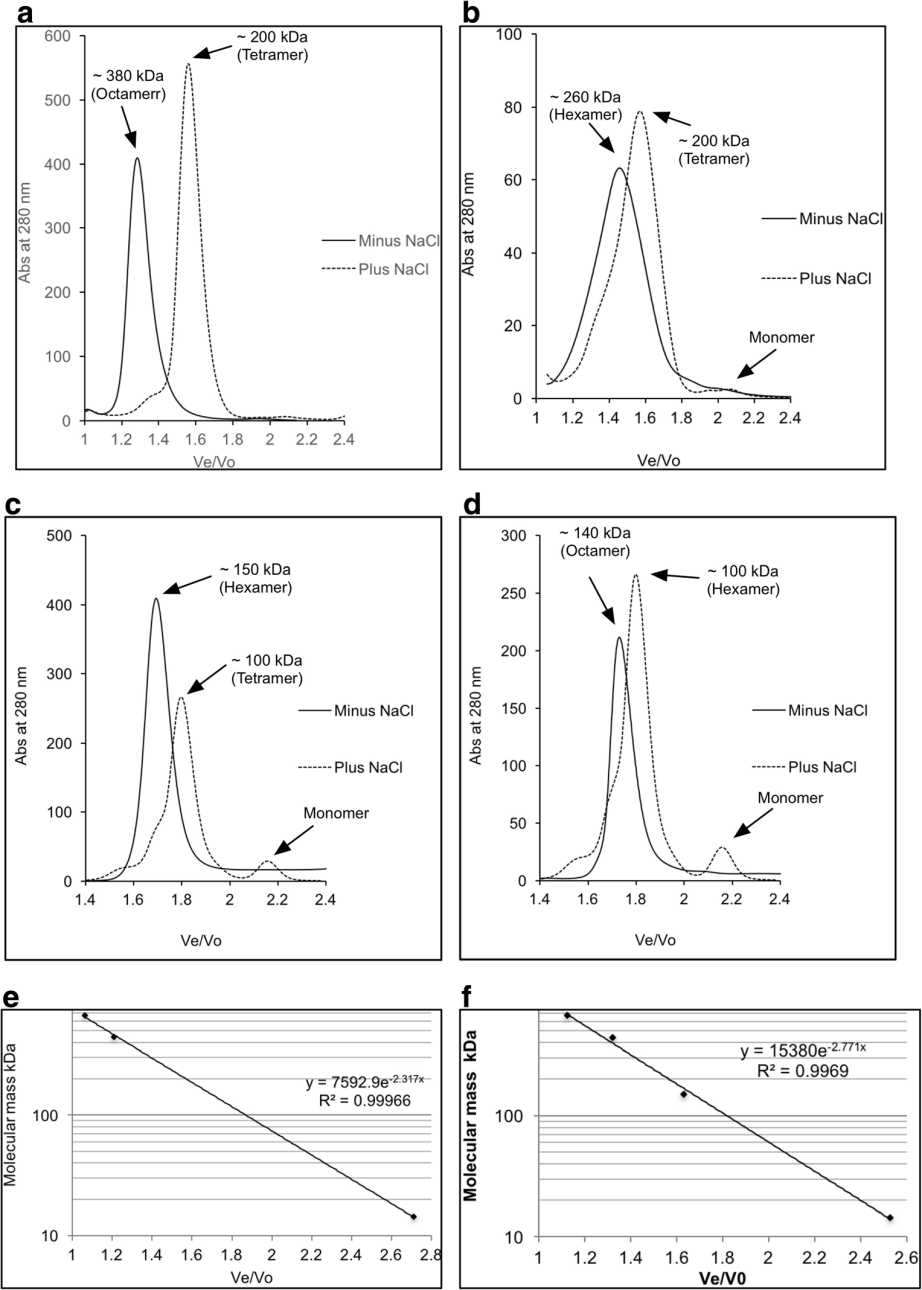
Separation of Hik2 oligomers by size-exclusion chromatography. Typical elution profiles of Hik2 proteins on a Superdex 200 column eluted with a buffer containing 20 mM Tris-HCl (pH 7.6) and 10 mM NaCl (solid line) or with 20 mM Tris-HCl (pH 7.6) and 500 mM NaCl (dotted line). **a** Syn_Hik2F. **b** Ther_Hik2F. **c** Ther_Hik2T. **d** Ther_DHp. The positions of hexamers, tetramers and octamers are shown. **e**, **f** Calibration curves (**e** 10 mM NaCl. **f** 500 mM NaCl) of the Superdex 200 using standard proteins of known molecular mass: apoferritin (443 kDa), alcohol dehydrogenase (150 kDa) and carbonic anhydrase (29 kDa). Blue dextran (2000 kDa) was used to determine the void volume (Vo). Ve is the effluent volume. On the *y*-axis, the base-ten logarithm of the protein molecular mass is shown, and on the *x*-axis, Ve/Vo is shown

On a Superdex 200 column calibrated with a buffer at low NaCl concentration (10 mM), the full-length *Synechocystis* and *Thermosynechococcus* and the truncated *Thermosynechococcus* Hik2 proteins eluted as hexamers with apparent molecular masses of 380, 260 and 150 kDa, respectively (Fig. 6a–c, solid lines). In the presence of NaCl at 500 mM final concentration, the apparent molecular mass was shifted from 380 to 200 kDa for *Synechocystis* Hik2, from 260 to 200 kDa for *Thermosynechococcus* Hik2 and from 150 to 100 kDa for the truncated *Thermosynechococcus* Hik2, corresponding to tetrameric forms (Fig. 6a–c, broken lines). These results may be consistent with those obtained from cross-linking experiments (Fig. 5b), provided that one assumes that contributions from tetramers to overlapping bands are difficult to resolve in the samples untreated with 500 mM NaCl. Since the DHp domain of the histidine kinase is important for its dimerisation activity, we explored the role of the DHp domain in higher-order oligomeric formation. Figure 6d shows that the DHp domain of *Thermosynechococcus* formed high-order oligomers that may be octamers in a buffer at low NaCl concentration and that the octamers converted to hexamers upon treatment with NaCl at 500 mM. It may be concluded that the DHp domain is of central importance for the salt-sensing activity of Hik2. The oligomeric states observed for the DHp domain are different from those seen for the full-length proteins, and it is possible that these are controlled by additional interactions involving other domains. It was noted that oligomeric states equivalent to the full-length proteins were only observed for the truncated *Thermosynechococcus* Hik2 (Thermo_Hik2T) form; thus, it is possible that the CA domain may play a role in defining the Hik2 oligomeric state.

### Transmission electron microscopy and single-particle analysis of negatively stained Hik2

**Fig. 7:**
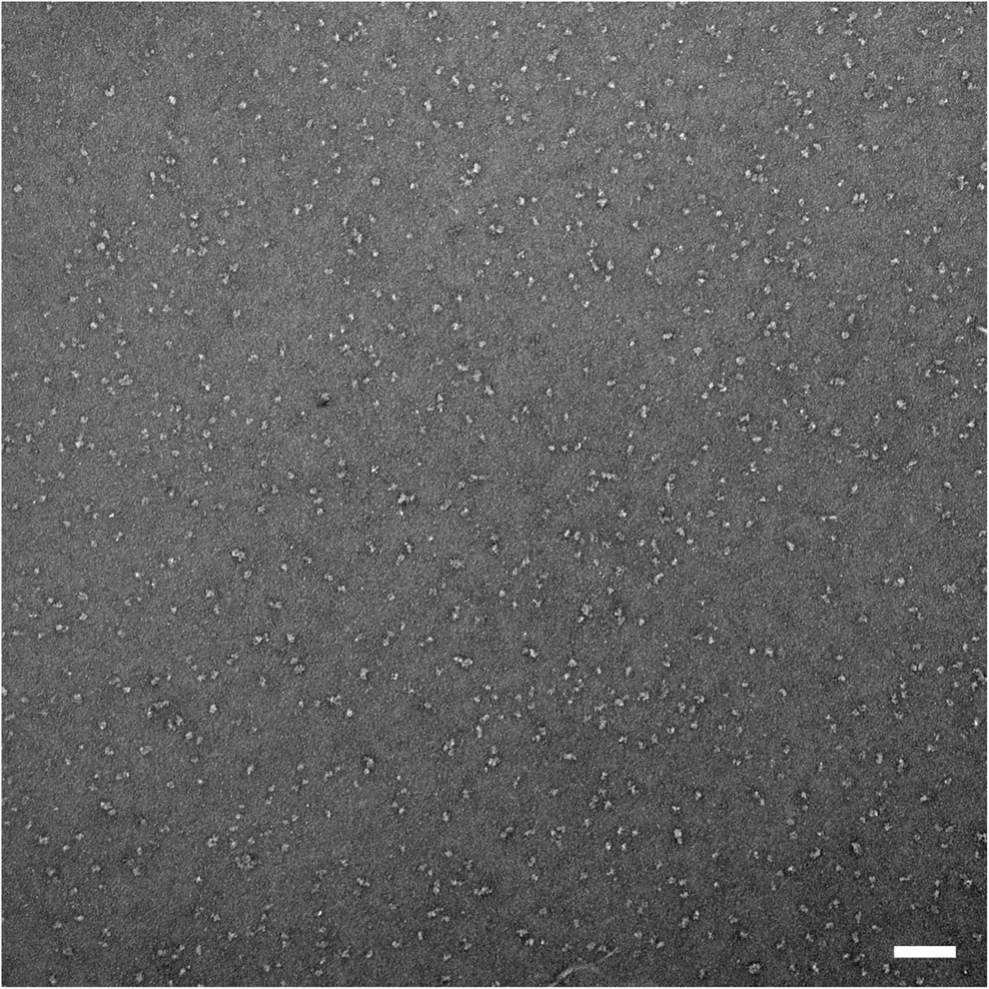
Characteristic micrograph of a negatively stained *Synechocystis* Hik2 protein sample. A typical micrograph as observed by TEM at × 80,000 magnification. Bar represents 100 nm

**Fig. 8:**
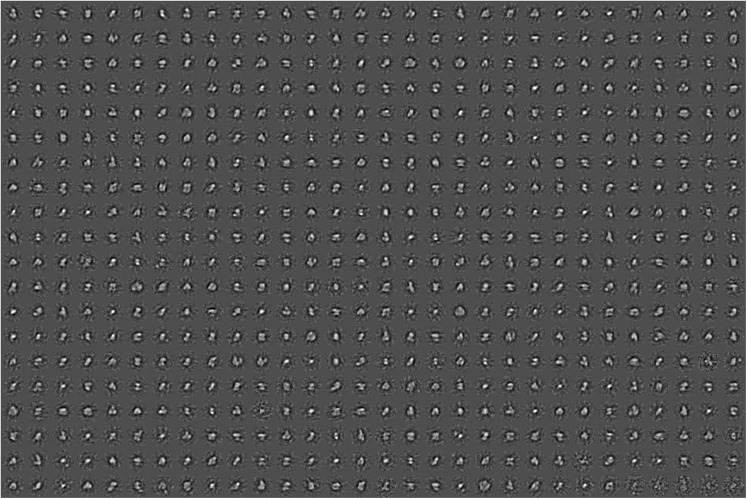
Structural heterogeneity of the Hik2 single-particle TEM-derived data set. The structural heterogeneity was revealed by relaxing the classification constraints, presenting all possible single particles automatically particle-picked from the micrographs as 600 characteristic views (class averages). Each boxed side of a single characteristic view represents 382 Å

**Fig. 9:**
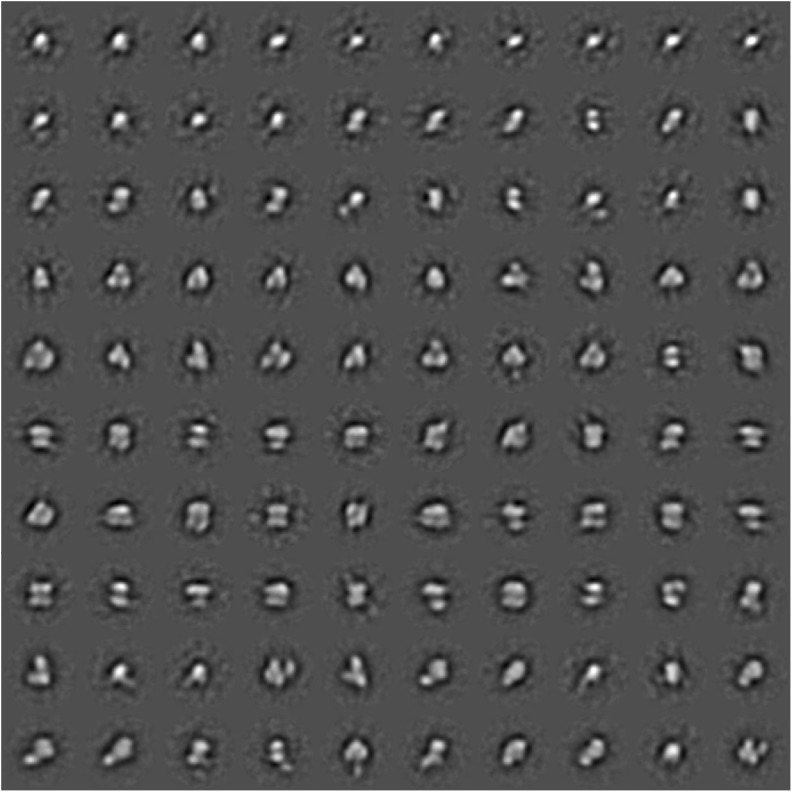
Classification of the Hik2 single-particle TEM-derived data set. After four rounds of iterative refinements, the data set was classified into 100 characteristic views (class averages). These views were re-ordered by visual inspection into oligomers of increasing diameter. Statistical analysis of this classification (see Table 3) revealed that these views represent 11,371 particles, which were subsequently attributed to oligomers ranging from monomers to double trimers (hexamers) and stacked/double tetramers (octamers). The side of each box, within which individual characteristic views are floated, represents 382 Å in length; thus, a typical monomer is ~ 80 Å in diameter and the largest views are ~ 180 Å in the long axis

**Fig. 10:**
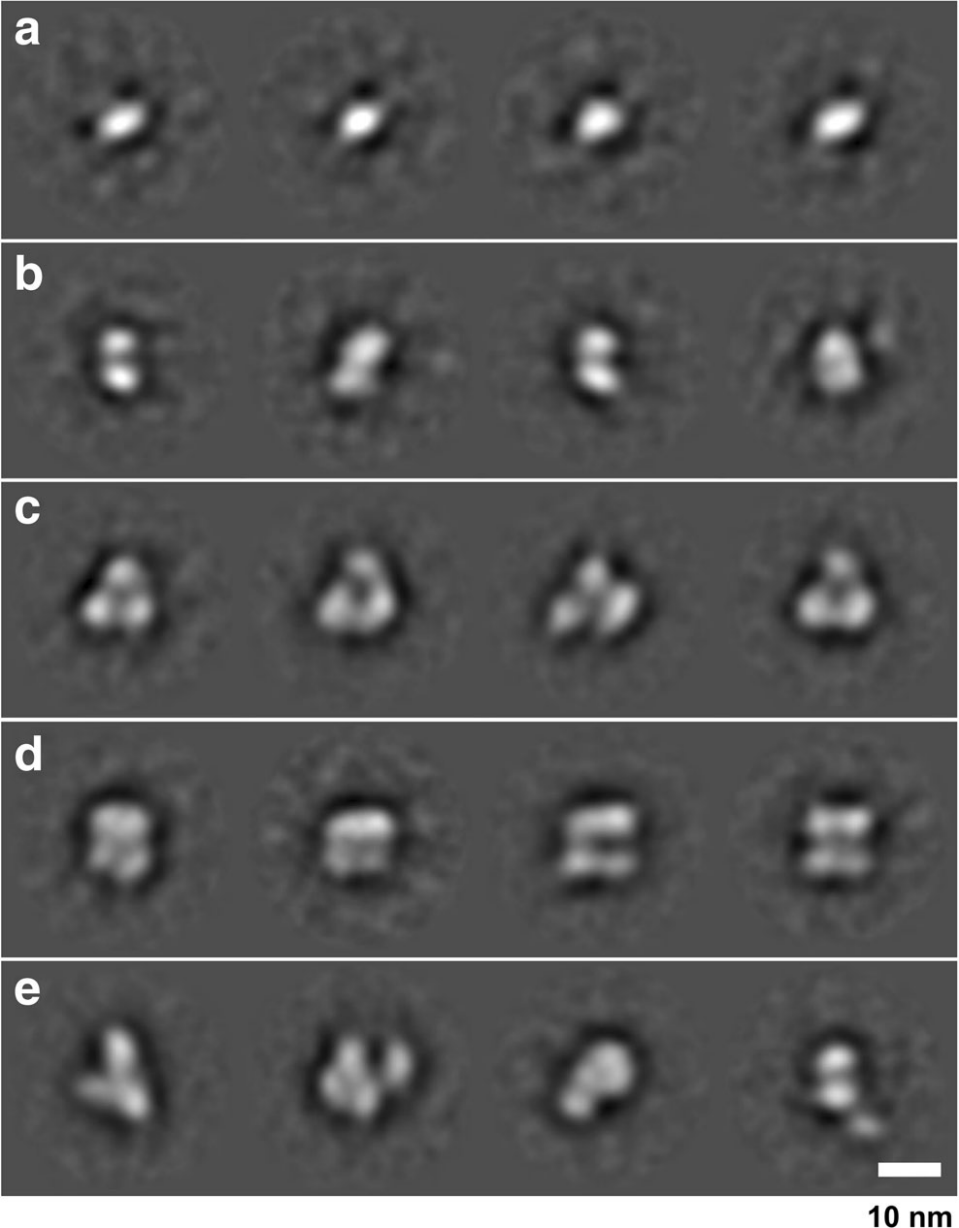
Single-particle averages of negatively stained samples imaged by transmission electron microscopy (TEM). After the image-processing techniques of multivariate statistical analysis and subsequent averaging, a series of Hik2 protein complex subpopulations or families were observed by TEM of negatively stained samples. A comparison of this figure with Table 3 provides a detailed statistical outcome for each subpopulation. Four representative averages for each family are shown. These are attributed to oligomeric states of **a** monomers, **b** dimers, **c** trimers or potential double trimers (hexamers) in projection, **d** tetramers or potential double trimers (hexamers) or double tetramers (octamers) in side elevation and **e** characteristic views of the remaining family of broken complexes or those with visible extraneous density. The *Synechocystis* sp. PCC 6803 Hik2 monomer has a molecular mass of ~ 50 kDa. Protein is white and the stain black. The scale bar is 10 nm

Five independent samples were negatively stained and imaged using a JEOL 1230 TEM equipped with a 2k Olympus Morada CCD camera system, and those micrographs that displayed the highest quality (41 from a total of 201, see ‘Materials and methods’ section) were carried forward for single-particle image analysis. Figure 7 is a micrograph of a negatively stained *Synechocystis* Hik2 protein sample as observed by TEM at × 80,000 magnification and typical for those used in the single-particle image-averaging analysis. The high contrast produced by the uranyl acetate stain allowed for the visual inspection of protein complexes. A variety of different sizes and orientations were observed. A data set of 13,341 individual protein complex images was built and subjected to reference-free alignment and multivariate statistical classification using Imagic-5 software. The spread of oligomeric states, i.e. structural heterogeneity, may be appreciated by relaxing the classification constraints so that the TEM-derived data set is represented as 600 class averages after four rounds of iterative refinement (Fig. 8). After four rounds of iterative refinement, unassigned particles were excluded leaving 11,371 particles that were classified into 100 class averages, or ‘characteristic views’ (Fig. 9 and Table 3). Each row of Fig. 10 depicts four of these characteristic views for each of the different oligomeric complex families assigned subjectively: monomers, dimers, trimers, double dimers or hexamers and broken or ambiguous density, respectively. It cannot be ruled out that the oligomers shown in Fig. 10b, c are double dimers (dimers of dimers) and double trimers (trimers of dimers), respectively, viewed in projection. Similarly, it is possible that the oligomers shown in Fig. 10c are double trimers or double tetramers (dimers of tetramers) viewed in side elevation.

**Table 3:** Single-particle image processing statistics relating to the characteristic views (averages) presented in Fig. 9 (100 averages), the final classification of the data set derived from micrographs of which Fig. 7 is typical. Each number below refers to the single particles present in each average; e.g. the first monomer average contains 107 particles

Monomers	Dimers	Trimers	Double dimers/hexamers	Broken or extraneous		
107	82	106	86	289		
133	111	152	164	94		
129	138	137	83	112		
124	109	197	95	107		
95	88	102	78	98		
127	128	213	182	90		
85	129	111	47	122		
90	96	164	68	141		
121	125	117	202	121		
99	117	119	181	91		
87	102	168	92	128		
106	94	102	169	127		
81	117	143	124	123		
107	85	165	161	129		
	91	100	85	84		
	117	57	47	74		
	99	92	133	111		
			67	76		
			115	105		
			115	140		
			70	103		
			114	99		
			71			
			70			
			69			
			113			
			83			
			125			
			82			
			98			
14 avgs	17 avgs	17 avgs	30 avgs	22 avgs	Avgs 100=	classums_100_4c.img
1491	1828	2245	3189	2564	11,317	Classified particles
13%	16%	20%	28%	23%	100%	
					2024	Junk, removed
				Avgs = averages	13,341	Total, 41 micrographs

## Discussion

It has been suggested that membrane-bound and soluble histidine kinases are homodimeric in their functional forms (Bilwes et al. 1999; Marina et al. 2005; Surette et al. 1996) and that some histidine kinases interconvert between an inactive monomer and an active dimer (Surette et al. 1996). Although histidine kinases are also reported to exist in higher-order oligomeric states, these states seem to be autokinase-inactive. The exception is the hybrid histidine kinase ExsG, which has been shown to be active as a hexamer (Wojnowska et al. 2013). The work presented here was directed at the elucidation of the oligomeric states of the soluble cyanobacterial Hik2 protein. Using chemical cross-linking, size-exclusion chromatography profiles and transmission electron microscopy, we find that the full-length Hik2 protein exists as tetramers, hexamers and other higher-order oligomers (Figs. 4, 6 and 10). Further analysis of the oligomeric states of Hik2 using truncated forms of the protein revealed that the oligomeric state of Hik2 is controlled by its kinase domain (Fig. 6c, d) and that the presence of NaCl at 500 mM converts the hexamer into a tetramer (Figs. 5b and 6), rendering the kinase inactive. Furthermore, the Hik2 protein is not always present in its full-length form and may be truncated in certain photosynthetic organisms (Table 2).

The occurrence of Hik2 in different forms implies that it may have multiple functions, some specific to certain species. The class I Hik2 protein homologues, which are found in chloroplasts and cyanobacteria, have a fully conserved GAF domain as their sensor domain and may therefore be able to sense a variety of signals. The class II Hik2, found only in marine cyanobacteria, has lost the GAF sensor domain completely (Table 2 and Fig. 2). Based on the results in Fig. 6c, d, the activity of this class II Hik2 may be regulated through its kinase domain. Class II Hik2 are found only in three cyanobacterial species: *Gloeobacter violaceus* PCC 7421, *Synechococcus* sp. JA-2-3B'a(2-13) and *Synechococcus* sp. JA-3-3Ab. Current phylogenetic trees indicate that these three cyanobacteria diverged very early from other cyanobacteria (Gupta 2009). It is therefore possible that the ancestral Hik2 protein lacked a sensor domain and later acquired the GAF sensor domain through gene fusion or acquisition. Alternatively, the sensor domain of class II Hik2 within those three cyanobacteria might have been lost after they diverged from a sensor domain-containing common ancestor.

The activity of histidine kinases is modulated by environmental cues through signal-induced conformational changes (Bhate et al. 2015; Mechaly et al. 2014; Wang et al. 2013). Chemical cross-linking and size-exclusion chromatography were utilised in order to understand the effect of salt stress on the oligomeric state and activity of Hik2. Both techniques revealed that the Hik2 protein complex exists predominantly as tetrameric, hexameric and other higher-order oligomeric forms (Figs. 4 and 6). Monomers are visible in Fig. 4a, b, lane 3. However, salt treatment decreased the monomeric forms of Hik2 (Fig. 5b, lane 4). Unlike the histidine kinases CheA (Surette et al. 1996), DcuS (Scheu et al. 2010), ArcB (Georgellis et al. 2001; Malpica et al. 2004) and RegB (Swem et al. 2003), the dimeric form of Hik2 was not detected, indicating that higher-order oligomers are the stable forms. Furthermore, the higher-order oligomeric states of Hik2 were functionally active (Fig. 5a). Treatment of Hik2 with NaCl to give a concentration of 500 mM converted the monomer, hexamer and octamer into the tetramer (Fig. 5b, lane 4, and Fig. 6). Results obtained from cross-linking (Fig. 5) and size-exclusion chromatography (Fig. 6) indicate that the inactivation mechanism of Hik2 involves the conversion of the higher-order oligomers into the tetramer. What remains to be clarified, however, is how it is possible that tetramers in Hik2 samples that were not treated with 500 mM NaCl exhibit autokinase activity, suggesting that these tetramers are structurally different from the inactive tetramers that form at elevated NaCl concentrations. Another possibility is that phosphorylated tetramers and monomers form only from autophosphorylated hexamers; in other words, only the hexamers are active in autophosphorylation.

The sensor domain of histidine kinases is usually required to detect signals, the exception being EnvZ, which was shown to receive signal through its DHp domain (Wang et al. 2012). It is therefore likely that Hik2 employs a DHp-based signal perception mechanism for its salt-sensing activity. We tested this possibility using three different variants of the Hik2 protein. Our result showed that the truncated forms of Hik2, consisting of the core kinase domain or DHp domain alone, were both present as a higher-order oligomer and that treatment with 500 mM NaCl converted them into the lower form of oligomer. We conclude that that the salt-sensing activity of Hik2 is confined to its DHp domain. In addition to salt, the GAF domain of Hik2, which is found in class I Hik2s, might be involved in perceiving additional signal(s) such as redox or it might bind small ligand(s) required to regulate its autophosphorylation (Allen 1993b). Indeed, the Hik2 homologue in higher plants has been shown to bind the PQ analogue DBMIB with a *K*_d_ value similar to that of other quinine-binding proteins (Puthiyaveetil et al. 2013); it also forms a quinone adduct (Ibrahim et al. 2016b).

**Fig. 11:**
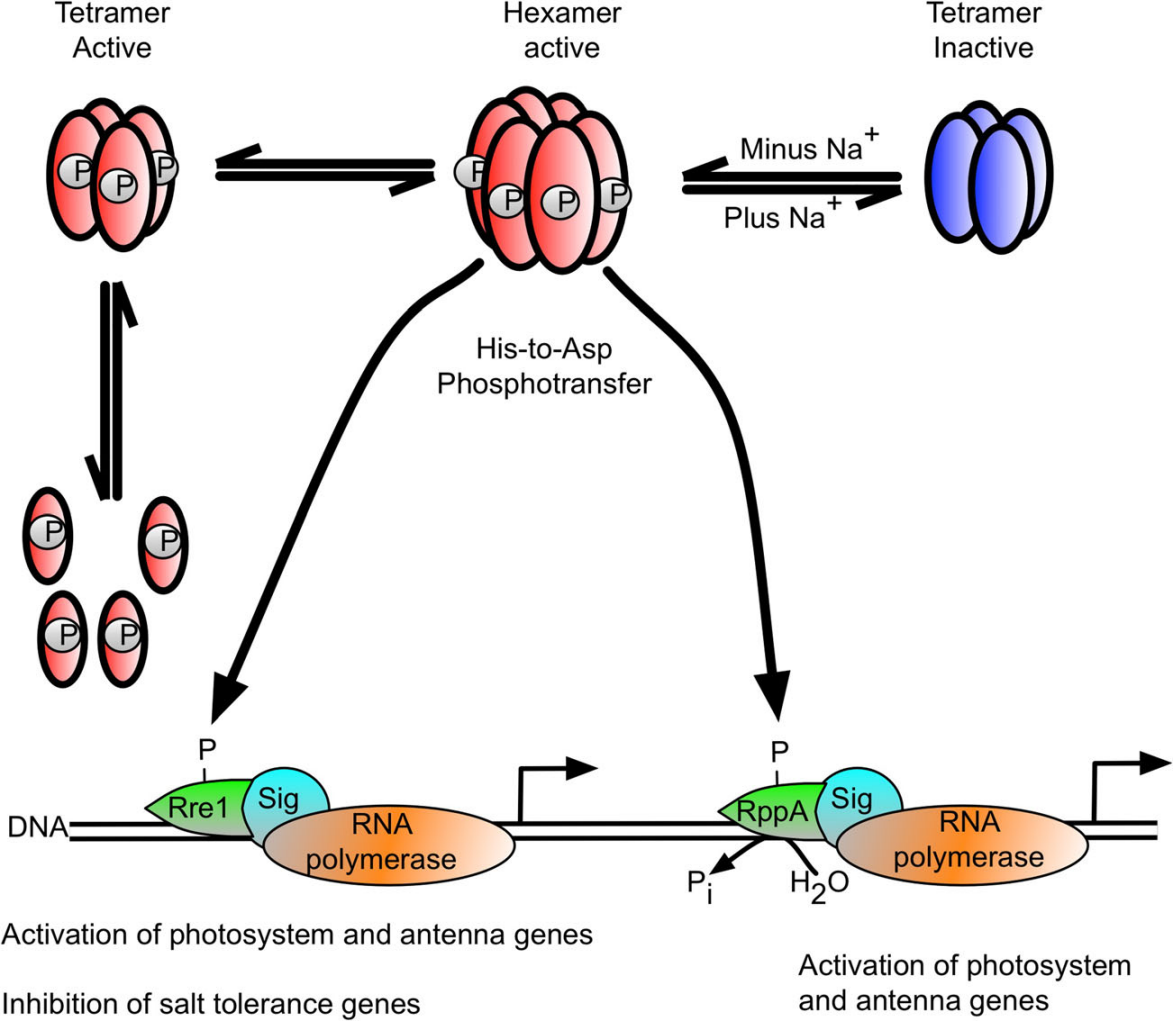
Proposal for a Hik2-based signal transduction pathway in cyanobacteria. The hexameric form of Hik2 is autokinase-active, and the oligomeric state of Hik2 is regulated by signals from Na^+^ and the photosynthetic electron transport chain. The active hexameric form of Hik2 is converted to an inactive tetramer upon salt stress

Figure 11 proposes a working model of signal perception mechanism of Hik2. In the absence of increased salt concentration or redox stresses, the Hik2 is autokinase-active and transfers phosphoryl groups to the response regulators Rre1 and RppA, while Phospho-Rre1 activates genes coding for light-harvesting phycobiliproteins. However, in the presence of salt/osmotic stress, the active hexameric form of Hik2 is rearranged into the inactive tetrameric form. Rre1 and RppA, therefore, remain in their unphosphorylated states. As a result, Rre1 can no longer act as a repressor of salt/osmotic tolerance genes, in turn releasing the repression of their transcription. We are unable to determine the precise symmetry of the oligomeric forms described here. For example, the hexamers could be either trimers of dimers of 32-point group symmetry or hexamers of hexagonal symmetry (point group 6). Similarly, the tetramers could be dimers of dimers (point group 22), or tetramers of tetragonal symmetry (point group 4), and tetragonal tetramers may assemble into octamers as dimers of tetramers (point group 42). Conversion between hexamers and tetramers is likely to involve only oligomers of the aforementioned symmetries. This conversion between oligomers would be possible if conformations of the subunits change during the transition. The molecular basis of a mechanism by which elevated Na^+^ concentrations trigger recombination of hexamers into tetramers could therefore be twofold. Na^+^ may interfere with salt bridges that stabilise intersubunit interfaces at low salt concentrations. In addition, Na^+^ may induce conformational changes in the protein subunits thus leading to the disruption of intersubunit interactions in the hexamers and favouring the formation of new protein-protein interfaces that result in inactive tetramers.

Hik2 is of special interest because of the presence of its homologue in all known cyanobacteria as well as in chloroplasts (Table 2). In eukaryotes, the homologue of Hik2 is termed CSK, a protein that is encoded in the nucleus and synthesised in the cytosol for post-translational import and processing in chloroplasts (Puthiyaveetil et al. 2012; Puthiyaveetil et al. 2008). CSK couples the redox state of the photosynthetic electron transport chain to chloroplast gene transcription (Puthiyaveetil and Allen 2008) by acting on plastid transcriptional regulators (Puthiyaveetil et al. 2013; Puthiyaveetil et al. 2010). It will be important to determine whether the oligomerisation that we propose as the basis of regulation by sodium ions is also a mechanism that extends to redox regulatory control of the activity of all Hik2 proteins, including CSK. The oligomeric states of Hik2 and CSK may determine their sensitivity and the amplification gain (Koshland et al. 1982) of the cell’s response to changes in both sodium ion concentration and redox state of photosynthetic electron carriers.

## References

[CR1] Allen JF (1993). Control of gene expression by redox potential and the requirement for chloroplast and mitochondrial genomes. J Theor Biol.

[CR2] Allen JF (1993). Redox control of transcription: sensors, response regulators, activators and repressors. FEBS Lett.

[CR3] Allen JF (2015). Why chloroplasts and mitochondria retain their own genomes and genetic systems: colocation for redox regulation of gene expression. Proc Natl Acad Sci USA.

[CR4] Allen JF (2017). The CoRR hypothesis for genes in organelles. J Theor Biol.

[CR5] Altschul SF, Gish W, Miller W, Myers EW, Lipman DJ (1990). Basic local alignment search tool. J Mol Biol.

[CR6] Ashby MK, Houmard J (2006). Cyanobacterial two-component proteins: structure, diversity, distribution, and evolution. Microbiol Mol Biol Rev.

[CR7] Bhate MP, Molnar KS, Goulian M, DeGrado WF (2015). Signal transduction in histidine kinases: insights from new structures. Structure.

[CR8] Bilwes AM, Alex LA, Crane BR, Simon MI (1999). Structure of CheA, a signal-transducing histidine kinase. Cell.

[CR9] Cai SJ, Khorchid A, Ikura M, Inouye M (2003). Probing catalytically essential domain orientation in histidine kinase EnvZ by targeted disulfide crosslinking. J Mol Biol.

[CR10] Duanmu DQ (2014). Marine algae and land plants share conserved phytochrome signaling systems. Proc Natl Acad Sci U S A.

[CR11] Filippou PS, Kasemian LD, Panagiotidis CA, Kyriakidis DA (2008). Functional characterization of the histidine kinase of the E. coli two-component signal transduction system AtoS-AtoC. Biochim Biophys Acta.

[CR12] Georgellis D, Kwon O, Lin ECC (2001). Quinones as the redox signal for the Arc two-component system of bacteria. Science.

[CR13] Gupta RS (2009). Protein signatures (molecular synapomorphies) that are distinctive characteristics of the major cyanobacterial clades. Int J Syst Evol Microbiol.

[CR14] Heermann R, Altendorf K, Jung K (1998). The turgor sensor KdpD of Escherichia coli is a homodimer. Biochim Biophys Acta Biomembr.

[CR15] Hirose Y (2013). Green/red cyanobacteriochromes regulate complementary chromatic acclimation via a protochromic photocycle. Proc Natl Acad Sci USA.

[CR16] Ibrahim IM (2013). Biochemical characterisation of the cyanobacterial Hik2-Rre1 two-component regulatory system (Ph.D.).

[CR17] Ibrahim IM, Puthiyaveetil S, Allen JF (2016). A two-component regulatory system in transcriptional control of photosystem stoichiometry: redox-dependent and sodium ion-dependent phosphoryl transfer from cyanobacterial histidine kinase Hik2 to response regulators Rrel and RppA. Front Plant Sci.

[CR18] Ibrahim IM, Puthiyaveetil S, Khan C, Allen JF (2016). Probing the nucleotide-binding activity of a redox sensor: two-component regulatory control in chloroplasts. Photosynth Res.

[CR19] Kacprzak S, Njimona I, Renz A, Feng J, Reijerse E, Lubitz W, Krauss N, Scheerer P, Nagano S, Lamparter T, Weber S (2017). Intersubunit distances in full-length, dimeric, bacterial phytochrome Agp1, as measured by pulsed electron-electron double resonance (PELDOR) between different spin label positions, remain unchanged upon photoconversion. J Biol Chem.

[CR20] Kobayashi I, Watanabe S, Kanesaki Y, Shimada T, Yoshikawa H, Tanaka K (2017). Conserved two-component Hik34-Rre1 module directly activates heat-stress inducible transcription of major chaperone and other genes in Synechococcus elongatus PCC 7942. Mol Microbiol.

[CR21] Koshland DE, Goldbeter A, Stock JB (1982). Amplification and adaptation in regulatory and sensory systems. Science.

[CR22] Laemmli UK (1970). Cleavage of structural proteins during assembly of head of bacteriophage-T4. Nature.

[CR23] Lomant AJ, Fairbanks G (1976). Chemical probes of extended biological structures: synthesis and properties of the cleavable protein cross-linking reagent [35S]dithiobis(succinimidyl propionate). J Mol Biol.

[CR24] Malpica R, Franco B, Rodriguez C, Kwon O, Georgellis D (2004). Identification of a quinone-sensitive redox switch in the ArcB sensor kinase. Proc Natl Acad Sci USA.

[CR25] Marina A, Waldburger CD, Hendrickson WA (2005). Structure of the entire cytoplasmic portion of a sensor histidine-kinase protein. EMBO J.

[CR26] Mechaly AE, Sassoon N, Betton JM, Alzari PM (2014). Segmental helical motions and dynamical asymmetry modulate histidine kinase autophosphorylation. PLoS Biol.

[CR27] Nagano S, Scheerer P, Zubow K, Michael N, Inomata K, Lamparter T, Krauss N (2016). The crystal structures of the N-terminal photosensory core module of agrobacterium phytochrome Agp1 as parallel and anti-parallel dimers. J Biol Chem.

[CR28] Noack S, Michael N, Rosen R, Lamparter T (2007). Protein conformational changes of agrobacterium phytochrome Agp1 during chromophore assembly and photoconversion. Biochemistry-Us.

[CR29] Pan SQ, Charles T, Jin SG, Wu ZL, Nester EW (1993). Preformed dimeric state of the sensor protein vira is involved in plant-agrobacterium signal-transduction. Proc Natl Acad Sci USA.

[CR30] Puthiyaveetil S, Allen JF (2008). Transients in chloroplast gene transcription. Biochem Biophys Res Commun.

[CR31] Puthiyaveetil S, Allen JF (2009). Chloroplast two-component systems: evolution of the link between photosynthesis and gene expression. Proc R Soc B Biol Sci.

[CR32] Puthiyaveetil S, Ibrahim IM, Allen JF (2012). Oxidation-reduction signalling components in regulatory pathways of state transitions and photosystem stoichiometry adjustment in chloroplasts. Plant Cell Environ.

[CR33] Puthiyaveetil S, Ibrahim IM, Allen JF (2013). Evolutionary rewiring: a modified prokaryotic gene regulatory pathway in chloroplasts. Philos Trans R Soc Lond B Biol Sci.

[CR34] Puthiyaveetil S, Ibrahim IM, Jelicić B, Tomasić A, Fulgosi H, Allen JF (2010). Transcriptional control of photosynthesis genes: the evolutionarily conserved regulatory mechanism in plastid genome function. Genome Biol Evol.

[CR35] Puthiyaveetil S, Kavanagh TA, Cain P, Sullivan JA, Newell CA, Gray JC, Robinson C, van der Giezen M, Rogers MB, Allen JF (2008). The ancestral symbiont sensor kinase CSK links photosynthesis with gene expression in chloroplasts. Proc Natl Acad Sci USA.

[CR36] Rockwell NC, Lagarias JC (2017). Phytochrome diversification in cyanobacteria and eukaryotic algae. Curr Opin Plant Biol.

[CR37] Sambrook J, Fritsch EF, Maniatis T (1989). Molecular cloning a laboratory manual second edition..

[CR38] Scheerer P, Michael N, Park JH, Nagano S, Choe HW, Inomata K, Borucki B, Krauß N, Lamparter T (2010). Light-induced conformational changes of the chromophore and the protein in phytochromes: bacterial phytochromes as model systems. ChemPhysChem.

[CR39] Scheu PD, Liao YF, Bauer J, Kneuper H, Basche T, Unden G, Erker W (2010). Oligomeric sensor kinase DcuS in the membrane of Escherichia coli and in proteoliposomes: chemical cross-linking and FRET spectroscopy. J Bacteriol.

[CR40] Schultz J, Milpetz F, Bork P, Ponting CP (1998). SMART, a simple modular architecture research tool: identification of signaling domains. Proc Natl Acad Sci U S A.

[CR41] Skerker JM, Perchuk BS, Siryaporn A, Lubin EA, Ashenberg O, Goulian M, Laub MT (2008). Rewiring the specificity of two-component signal transduction systems. Cell.

[CR42] Stock AM, Robinson VL, Goudreau PN (2000). Two-component signal transduction. Annu Rev Biochem.

[CR43] Surette MG, Levit M, Liu Y, Lukat G, Ninfa EG, Ninfa A, Stock JB (1996). Dimerization is required for the activity of the protein histidine kinase CheA that mediates signal transduction in bacterial chemotaxis. J Biol Chem.

[CR44] Swem LR, Kraft BJ, Swem DL, Setterdahl AT, Masuda S, Knaff DB, Zaleski JM, Bauer CE (2003). Signal transduction by the global regulator RegB is mediated by a redox-active cysteine. EMBO J.

[CR45] Tang G, Peng L, Baldwin PR, Mann DS, Jiang W, Rees I, Ludtke SJ (2007). EMAN2: an extensible image processing suite for electron microscopy. J Struct Biol.

[CR46] van Heel M, Harauz G, Orlova EV, Schmidt R, Schatz M (1996). A new generation of the IMAGIC image processing system. J Struct Biol.

[CR47] Wang C, Sang J, Wang J, Su M, Downey JS, Wu Q, Wang S, Cai Y, Xu X, Wu J, Senadheera DB, Cvitkovitch DG, Chen L, Goodman SD, Han A (2013). Mechanistic insights revealed by the crystal structure of a histidine kinase with signal transducer and sensor domains. PLoS Biol.

[CR48] Wang LC, Morgan LK, Godakumbura P, Kenney LJ, Anand GS (2012). The inner membrane histidine kinase EnvZ senses osmolality via helix-coil transitions in the cytoplasm. EMBO J.

[CR49] Wojnowska M, Yan J, Sivalingam GN, Cryar A, Gor J, Thalassinos K, Djordjevic S (2013). Autophosphorylation activity of a soluble hexameric histidine kinase correlates with the shift in protein conformational equilibrium. Chem Biol.

